# Roles of target detection and behavioral response on pupil dilation and concurrent memory: An attentional boost study

**DOI:** 10.3758/s13414-025-03221-4

**Published:** 2026-02-23

**Authors:** Yi Ni Toh, Vanessa G. Lee

**Affiliations:** 1 Department of Psychology and Center for Cognitive Sciences, University of Minnesota, Minneapolis, MN USA; 2 Schepens Eye Research Institute of Mass Eye and Ear, Department of Ophthalmology, Harvard Medical School, Boston, MA USA

**Keywords:** Attentional boost effect, Pupil dilation, Dual-task performance, Attention and memory, Temporal orienting

## Abstract

The attentional boost effect (ABE) describes the counterintuitive phenomenon in which detecting and responding to behaviorally relevant targets improves the processing of unrelated stimuli presented at the same time. To better understand the mechanisms behind this dual-task enhancement, we compared behavioral and pupillary measures of the ABE, isolating the contributions of perceptual target detection and response requirements. Participants monitored a rapid serial presentation of a specific target letter (e.g., “E”), a large set of nontarget letters, and blank (baseline) trials in equal proportions. In one condition, they pressed a button for the target letter only; in another, for the nontarget letters. Experiment 1 assessed the ABE behaviorally by measuring memory for concurrently presented photographs. Experiment 2 assessed physiological responses via pupillometry during the same letter task (without image encoding). Memory was enhanced for target-paired images when participants responded to targets and showed a modest enhancement for both target- and nontarget-paired images when participants responded to nontargets. This is consistent with the idea that both perceptual and response goals modulate the ABE. Pupil dilation was also sensitive to both factors but was more strongly driven by response. These findings highlight the dual contributions of detection and response in producing the ABE.

**Supplementary Information:**

The online version contains supplementary material available at 10.3758/s13414-025-03221-4.

## Introduction

Daily activities often require us to rapidly identify and respond to important events while maintaining broader situational awareness. For example, a driver must monitor traffic signals while attending to pedestrians and other cars. The attentional boost effect (ABE) refers to the counterintuitive finding that detecting task-relevant targets can enhance memory and perception for concurrently presented, task-irrelevant stimuli (for review, see Swallow et al., 2022). In standard ABE paradigms, participants view a stream of background images while monitoring a rapid serial visual presentation (RSVP) and responding (e.g., via keypress) to a target-colored item. Although target detection increases attentional load, as shown in dual-task costs like the attentional blink (Duncan, 1980; Martens & Wyble, 2010; Raymond et al., 1992), target-paired images are typically remembered better than distractor-paired images. This effect has been replicated across a wide range of stimuli and tasks, yet its underlying mechanisms remain unclear. In this study, we aim to dissociate the effects of target detection and the need to respond, using both memory performance and pupil dilation as converging behavioral and physiological measures.

A leading theory, known as the temporal orienting account, attributes the ABE to phasic activation of the locus coeruleus–norepinephrine (LC–NE) system in response to target detection (Swallow et al., 2012, 2019, 2022; Yebra et al., 2019). When a target is detected, the LC generates a transient burst of norepinephrine (NE), which modulates widespread cortical networks and enhances sensory processing (Aston-Jones & Cohen, 2005). Functional MRI studies have linked LC activation to the ABE (Moyal et al., 2022), and pupil dilation, an indirect measure of LC activity (de Gee et al., 2017; Joshi et al., 2016; Murphy et al., 2014; Privitera et al., 2020), is greater for target trials than for nontarget trials, with pupil size predicting subsequent memory (Swallow et al., 2019; Yebra et al., 2019). These converging findings support the idea that LC–NE activity may underlie the ABE. However, because target trials involve both detection and response, it is unclear whether both components contribute to the effect. It remains an open question whether target detection and response have similar effects on memory and pupil dilation, as would be expected if both arise from LC–NE activity.

Swallow and Jiang (2013) proposed the dual-task interaction model, which attributes the ABE to target detection – that is, the decision that an RSVP stimulus matches the target template. This decision is thought to trigger a temporal orienting response that enhances processing of concurrently presented images. Other accounts emphasize later stages of processing, particularly the keypress response to the target. Yebra et al. (2019) referred to this as “motor-induced memory enhancement,” while Schonberg et al. (2014) referred to it as “cue approach” training. Still others have attributed the ABE to an intermediate stage—the need to respond—rather than the motor execution itself (Toh & Lee, 2022a, 2022b).

Empirical evidence to date supports both target detection and the need to respond as critical for inducing the ABE, while ruling out motor execution as a significant factor. First, the ABE occurs even without an overt motor response. Participants showed better memory for target-paired items when responses were delayed until the end of the RSVP sequence (Lin et al., 2010; Seitz & Watanabe, 2003) or when targets were counted silently (Mulligan et al., 2016; Swallow & Jiang, 2012; Toh & Lee, 2022b). Second, whereas responding to auditory targets enhances early visual activity, making random button presses does not (Swallow et al., 2012). These findings suggest that what drives the ABE is not motor execution but the cognitive operations leading up to it.

Toh and Lee (2022a, 2022b) refined this concept by distinguishing perceptual targets (stimuli that match a perceptual template) and response targets (stimuli that signal the need to respond). For example, a farmer sorting apples might look for signs of rot (perceptual targets) while packing away the good apples (response targets). The decision to act on an item as a response target is a distinct cognitive process that occurs before the physical act of packing. In most ABE paradigms, perceptual and response goals are aligned (e.g., pressing a key when a blue square appears), and this alignment typically produces a strong ABE. However, when participants are instructed to respond to nontarget (e.g., pressing a key for all letters except “T”), perceptual and response goals become misaligned. In such cases, both target and nontarget stimuli may trigger orienting responses, blurring the distinction between them and potentially weakening the ABE.

In a series of experiments, Toh and Lee (2022a, 2022b) showed that both perceptual and response goals shape the ABE. When participants responded only to the target letter “T,” memory for T-paired images was enhanced. But when they responded to all letters except “T,” the advantage disappeared. This finding suggests that response demands matter: alignment of perceptual and response goals produces an ABE, while misalignment abolishes it. In a third condition, participants pressed one button for “T” and another for non-T letters. Memory was enhanced for T-paired objects, suggesting that perceptual target detection alone is sufficient to elicit an ABE when response demands are held constant. To account for these findings, Swallow et al. (2022) updated the dual-task interaction model, attributing the ABE to the detection of stimuli that deviate from the default state—whether perceptual targets or response targets.

While these findings highlight the roles of perceptual target detection and the need to respond in the ABE, two critical questions remain. First, does the LC–NE system underlie both detection- and response-driven effects? In monkeys, phasic LC activation occurs prior to motor execution in go/no-go detection tasks (Aston-Jones et al., 1994), and the timing of LC activation tracks response latency as task difficulty increases (Rajkowski et al., 2004). However, these studies do not distinguish target detection from the need to respond. In humans, although LC imaging is possible using fMRI, its small size and proximity to cerebrospinal fluid pose challenges (Turker et al., 2021). Pupillometry offers an indirect but practical alternative, as pupil dilation is tightly coupled with LC activity (de Gee et al., 2017; Leong et al., 2021; Privitera et al., 2020; Swallow et al., 2019; Yebra et al., 2019). Studies have found a robust connection between pupil and phasic LC activity during arousal, surprise and decision making (Breton-Provencher et al., 2021; Hayat et al., 2020; Liu et al., 2017; Varazzani et al., 2015). Although pupil dilation is not a real-time one-on-one readout of the LC activity (Breton-Provencher & Sur, 2019; Joshi et al., 2016; Megemont et al., 2022; Yang et al., 2021), it is a valuable and commonly used indirect index of LC-NE engagement in cognitive tasks (Aston-Jones & Cohen, 2005; Clewett et al., 2020; Preuschoff et al., 2011).

Second, is the ABE best characterized as an absolute boost for target-paired stimuli, or as a relative boost resulting from inhibition of nontarget-paired stimuli? Some studies report that target-paired items are remembered better than both nontarget-paired and blank-baseline items, indicating an absolute enhancement (Lin et al., 2010; Swallow & Jiang, 2014). For example, Swallow and Jiang (2014) found that when the RSVP stream included a random mix of targets, nontargets, and blank trials, memory for target-paired images exceeded that for both distractor-paired and baseline images. In contrast, other studies using single-task baselines have found evidence consistent with a relative boost (Spataro et al., 2013). Spataro et al. (2013), for instance, compared a single-task condition (in which participants encoded a stream of words alone) with dual-task conditions in which some words were paired with a target color and others with a nontarget color. In explicit memory tests, target-paired words were remembered as well as single-task words, but better than distractor-paired words, indicating a relative, but not absolute, advantage. Still, some studies have reported a small but significant impairment for nontarget-paired images relative to baseline (Sisk & Lee, 2024), and others found only nontarget-related impairment without an advantage for target-paired images over blank baseline (Lee, 2023).

To address these questions, the present study manipulated perceptual and response goals, measured both memory and pupil responses, and included blank baseline trials for comparison. During encoding, participants viewed RSVP streams of letters and blanks overlaid on object images (Experiment 1) or on a blank background (Experiment 2). In each experiment, they were instructed to respond to either target or nontarget letters while ignoring blanks, allowing us to manipulate the alignment between perceptual and response goals. Blank trials, which were never associated with a response, served as a baseline. Experiment 1 assessed memory for target-, nontarget-, and blank-paired images. Experiment 2 measured pupil dilation in response to the same RSVP stimuli, isolating LC-related responses without interference from background images.

We tested two primary hypotheses. First, both perceptual and response goals contribute to the ABE. When goals align (respond to target), we expect enhanced memory and greater pupil dilation for target-paired items. When goals misalign (respond to nontarget), these enhancements should diminish. Second, if the ABE reflects an absolute boost, target-paired items should elicit stronger memory and pupil responses than both nontarget-paired and baseline trials. Alternatively, if nontarget inhibition contributes, nontarget-paired items may show reduced responses relative to baseline. Converging or diverging patterns across memory and pupil dilation will clarify whether these measures reflect a shared neuromodulatory mechanism—phasic LC–NE activation—or distinct cognitive processes.

## Experiment 1

This experiment includes a blank baseline to assess whether the ABE reflects target-related boost or distractor-related interference. We also examined how aligning or misaligning perceptual and response goals modulate the effect. Participants encoded a series of objects into memory while monitoring an RSVP stream of letters. The RSVP stream contained three trial types, randomly intermixed with equal probability: targets (a specific letter), nontargets (the other letters), and blank trials (no letters). In Experiment 1A, participants pressed a button for the target letter and withheld responses on other trials. In Experiment 1B, they responded to the nontarget letters and made no response to target or blank trials. We examined how the differing response demands in Experiments 1A and 1B affected the ABE, and whether target-paired images were remembered better than those paired with blanks.

Experiment 1 tested three competing hypotheses. First, if perceptual targets drive the ABE, memory for target-paired objects should be similarly enhanced in both Experiments 1A and 1B. Alternatively, the response hypothesis predicts that memory will be better for stimuli paired with the response, whether the response is to the search target (Experiment 1A) or to nontargets (Experiment 1B). Finally, if both perceptual and response goals influence the ABE, the effect should appear when the two goals align (Experiment 1A) but not when they misalign (Experiment 1B). The inclusion of blank trials allowed us to determine whether the observed boost reflected an absolute enhancement for target-paired stimuli or relative difference driven by nontarget inhibition.

### Method

#### Participants

##### Sample-size determination.

We predetermined a sample size of 36 participants per group (Experiments 1A and 1B) based on an a priori power analysis using G*Power (Faul et al., 2007). This analysis was based on a recent ABE study that also used objects as background images and included three trial types: target, nontarget, and blank (Sisk & Lee, 2024). Because we were interested in both the comparison between target- and nontarget-paired images (to assess the ABE) and the comparison between target-paired images and blank trials (to test for absolute vs. relative boost), our power analysis was based on findings from Sisk and Lee (2024). Using a sample of 44 participants, Sisk and Lee (2024) reported effect sizes of *d* = 1.33 for the target versus nontarget comparison and *d* = 0.61 for the target vs. blank comparison. Based on these values, a sample of 36 participants provides over 99% power to detect an ABE (target- vs. nontarget-paired images) and 94% power to detect a boost for target-paired images relative to the blank baseline (two-tailed tests, α =.05).

##### Participant characteristics.

Participants were recruited via Prolific.com, provided informed consent online, and completed the experiment on Pavlovia.org using their personal laptops or desktop computers. Eligibility criteria included being native English speakers, aged 18–45, having normal or corrected-to-normal vision, and no self-reported history of neurological or psychiatric disorders. Participants were compensated at a rate of $12 per hour. All study procedures were approved by the University of Minnesota Institutional Review Board.

Because Experiment 1 was conducted online, where shorter sessions are recommended to maintain engagement and data quality, we split the two response conditions into separate experiments and used a between-subjects design. In Experiment 1A (Respond to Target), 36 participants (17 women, 19 men; mean age = 31.1 years, *SD* = 7.4, range: 21–45 years) were included. Two additional participants were excluded due to duplicated participant numbers; in these cases, data from the later participants were excluded. In Experiment 1B (Respond to Nontarget), 36 participants (seven women, 29 men; mean age = 30.6 years, *SD* = 6.9, range: 20–43 years) were included. Three additional participants were excluded due to low detection accuracy falling below the prespecified cutoff of 75%, one was excluded due to duplicated participant numbers.

#### Equipment

Data collection was conducted on Pavlovia.org, an online platform integrated with PsychoPy (Peirce et al., 2019). The experiment was programmed in PsychoPy with JavaScript modifications for compatibility with Pavlovia.org. Condition files for PsychoPy were generated using MATLAB (https://www.mathworks.com/). The program automatically detected the participants’ device and launched only on desktop or laptop computers.

#### Material

##### **RSVP letters.**

The RSVP stimuli were white letters (Arial font, size 30) presented on a gray square background (40 × 40 pixels). These stimuli were also used in the pupillometry study (Experiment 2), necessitating us to control for luminance across stimuli. To this end, we implemented a data-driven selection procedure in MATLAB. For each of the 26 letters (Arial font, size 100), a grayscale image was generated and saved as a JPEG file (300 DPI). Luminance was quantified for each letter by calculating the mean pixel value of its grayscale representation using the “rgb2gray” function in MATLAB. Letters with extreme luminance values were identified as outliers based on the interquartile range (IQR; values falling outside 1.6 times the IQR from the median). This procedure led to the exclusion of six letters (“I,” “J,” “L,” “M,” “T,” “W”). From the remaining 20 letters, we selected the four target letters (“A,” “E,” “H,” “S”) whose luminance values were closest to the median luminance of this reduced set.

Each participant was randomly assigned one of these four letters as their designated target letter, with target letter assignment counterbalanced across participants. Nontarget letters were randomly selected from the remaining 19 letters (excluding the assigned target letter) and were not repeated within a block until all had been used once. This procedure ensured that the mean luminance of the nontargets was similar to that of the target, for any of the four targets assigned to participants.

##### Objects.

Objects were photographic images drawn from a database of 310 object categories compiled by Brady et al. (2008). From this set, we randomly selected 200 categories for use in Experiment 1. These were further divided into three subsets: 20 categories for practice trials, 90 categories for the encoding phase, and 90 novel categories reserved for the recognition test as foils. For each category used during encoding, one exemplar was selected as the encoded “old” object and the other as a within-category foil. In the recognition test, each encoded object was presented alongside three foils: (1) a within-category foil (a different exemplar from the same category) and (2) two novel foils (exemplars from categories not shown during encoding). All object images were presented against a white background. During encoding, images were presented at the center of the screen at 400 × 400 pixels. During the recognition test, the four choices were presented side-by-side in a horizontal row, each resized to 200 × 200 pixels. Object assignments to encoding, testing, and foil conditions, as well as their pairings with RSVP trial types (target, nontarget, blank), were fully counterbalanced across participants to minimize potential biases in stimulus memorability.

#### Design and procedure

##### **Practice phase.**

Prior to the main experiment, participants completed two practice tasks: a single-task target detection practice, followed by a dual-task practice session.

##### Single-task target detection practice.

Participants first performed a target detection task to familiarize themselves with the RSVP stream and response requirements. Participants were instructed to monitor an RSVP stream of letters and blank squares for their pre-assigned target letter (“A,” “E,” “H,” or “S”) counterbalanced across participants. In Experiment 1A, participants were instructed to press the space bar only when they detected their target letter, withholding responses to all other trials. In Experiment 1B, participants were instructed to press the space bar for any *letter* that was not their target letter, withholding responses to target letters and blank squares. Each trial in this practice task followed the same timing as the main experiment’s RSVP stream: a 200ms white noise mask, a 200ms presentation of a gray square (containing either a target letter, a nontarget letter, or a blank square, each with equal probability), and a 1.6-s white noise mask, resulting in a 2-s trial duration. The single-task practice ended once participants made five consecutive correct responses to the target.

##### Dual-task practice.

Next, participants completed a dual-task practice that was identical in structure to the encoding phase of the main experiment, requiring participants to perform the target detection task while also encoding object images that were superimposed behind the letter squares. Each trial in this dual-task practice also followed a 2-s duration, beginning with a 200-ms white noise mask, then a 200-ms presentation of the gray square (containing a letter or blank) overlaid on an object image that lasted by itself for an additional 300ms, and a 1.3-s white noise mask. Participants were told to prioritize letter detection but to pay attention to the object images as well. Participants in Experiment 1A responded to target letters; participants in Experiment 1B responded to nontarget letters. The practice phase ended when participants made five consecutive correct responses to the target. Object memory was not tested during practice, and participants were told to remember the object images without being informed about the upcoming recognition test format.

##### Experimental phase.

**Fig. 1:**
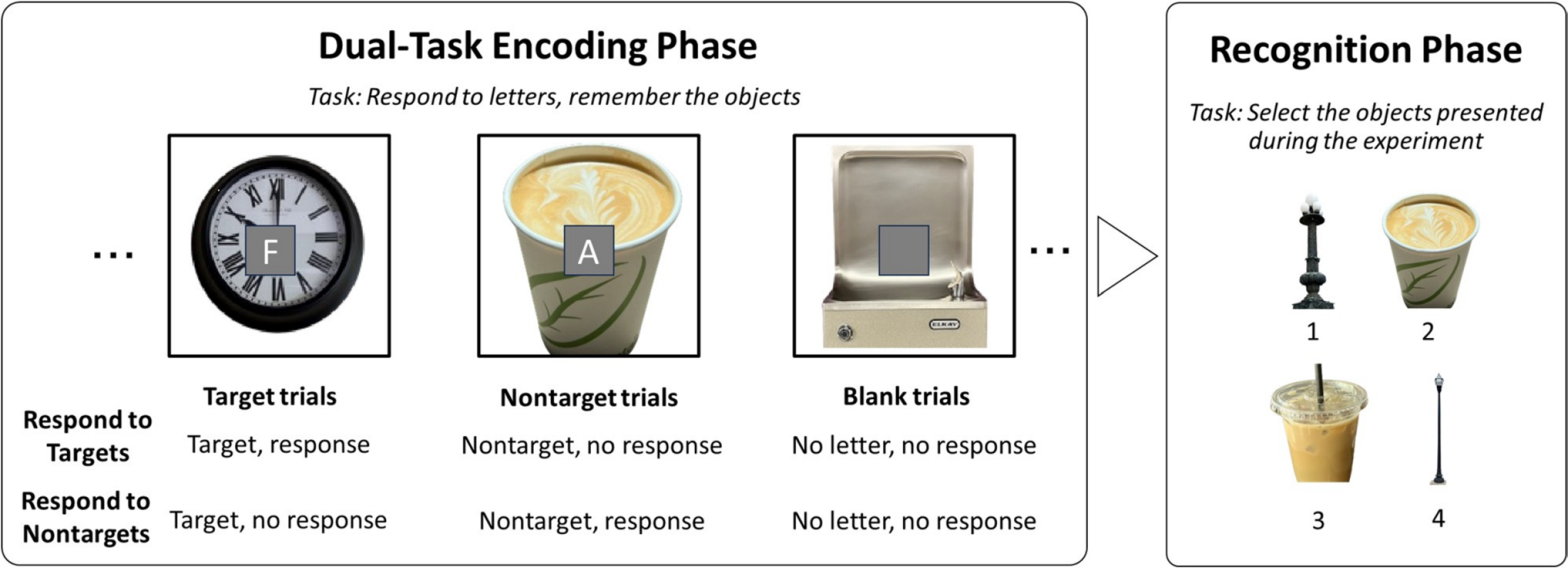
Study design for Experiments 1A and 1B (not to scale). Left panel: Dual-task encoding phase. Participants performed a rapid serial visual presentation (RSVP) letter detection task while simultaneously encoding object images. In Experiment 1A (Respond to Target), participants pressed the space bar when they saw a pre-assigned target letter (such as the letter *F*) and withheld responses to all other letters and blank trials. In Experiment 1B (Respond to Nontarget), participants pressed the space bar for any nontarget letter and withheld responses for target letters and blank trials. Target, nontarget, and blank trials occurred with equal frequency in both experiments. Right panel: Recognition phase**.** Participants completed a four-alternative forced-choice memory test, selecting the encoded object from a set of four choices: the encoded object, a within-category foil, and two novel foils. (Color figure online)

The main experiment consisted of a dual-task encoding phase immediately followed by a four-alternative forced-choice (4AFC) recognition test (see Fig. 1 for a study design overview). 

##### ***Dual-task encoding phase (***Fig. 1***, left).***

This was structurally identical to the dual-task practice. Each block consisted of 90 trials, with an equal number of target-paired, nontarget-paired, and blank-paired objects (30 trials each). Participants completed three blocks, totaling 270 trials. Participants received feedback on their letter detection accuracy at the end of each block. To ensure that memory performance was above chance, the 90 unique objects were each presented once per block, with trial order randomized within each block and reshuffled across blocks. This resulted in each object being presented three times over the course of the encoding phase.

##### ***Recognition phase (***Fig. 1***, right).***

Participants completed a 4AFC recognition test to assess object memory following the encoding phase. On each trial, four object images were displayed in a horizontal row, spaced 210 pixels apart, with numerical labels (1–4) shown below each image. Each recognition trial presented four choices: the previously encoded object, a within-category foil, and two novel foils. The positions of the four objects were randomized across trials. Participants selected the number (1–4) corresponding to the object they believed had appeared during encoding. Correct responses were followed by the word “Correct!” displayed in green for 500 ms at the center of the screen; incorrect responses received no feedback. The recognition test consisted of 90 trials, corresponding to the 90 objects shown during encoding.

We employed a 4AFC recognition task as it provides a sensitive measure of recognition memory and allows for the assessment of both category and exemplar memory (Sisk & Lee, 2022). Complete datasets for all experiments are available on the Open Science Framework (OSF) at (https://osf.io/7a8v6/).

### Results

#### Letter detection

##### **Respond to target.**

Participants responded to target letters 97.4% (*SE* = 0.6%) of the time, with a mean response time (RT) of 510 ms (*SE* = 13 ms). They false alarmed to nontarget letters 1.7% (*SE* = 0.3%) of the time and to blank squares 0.3% (*SE* = 0.1%) of the time, with significantly higher false-alarm rate for nontarget letters than blank squares, *t*(35) = 5.10, *p* <.001, Cohen’s *d* = 0.85, indicating greater difficulty in withholding responses to nontargets compared with blank squares.

##### Respond to nontarget.

When the response rule was reversed, participants responded to nontarget letters 97.4% (*SE* = 0.7%) of the time, with a mean RT of 619ms (*SE* = 16 ms). They false alarmed to target letters 4.4% (*SE* = 0.5%) of the time and to blank squares 1.0% (*SE* = 0.2%) of the time, with significantly higher false-alarm rates to target letters compared with blank trials, *t*(35) = 5.75, *p* <.001, Cohen’s *d* = 0.96, suggesting that blank trials imposed lower attentional demands than target letters.

##### Combined analysis of trial condition and response demands.

When hit and false-alarm rates were combined into a *d′* measure, performance in Respond to Target (*M* = 4.51, *SE* = 0.10) was significantly higher than in Respond to Nontarget (*M* = 4.17, *SE* = 0.11), *t*(70) = 2.27, *p* =.02, Cohen’s *d* = 0.53. Additionally, mean RT was significantly shorter in Respond to Target than in Respond to Nontarget, *t*(70) = 5.34, *p* <.001, Cohen’s *d* = 1.26. These results suggest that the task was easier when the target letter aligned with the key press response, compared with when the response required withholding for targets.

#### Object Memory

**Fig. 2:**
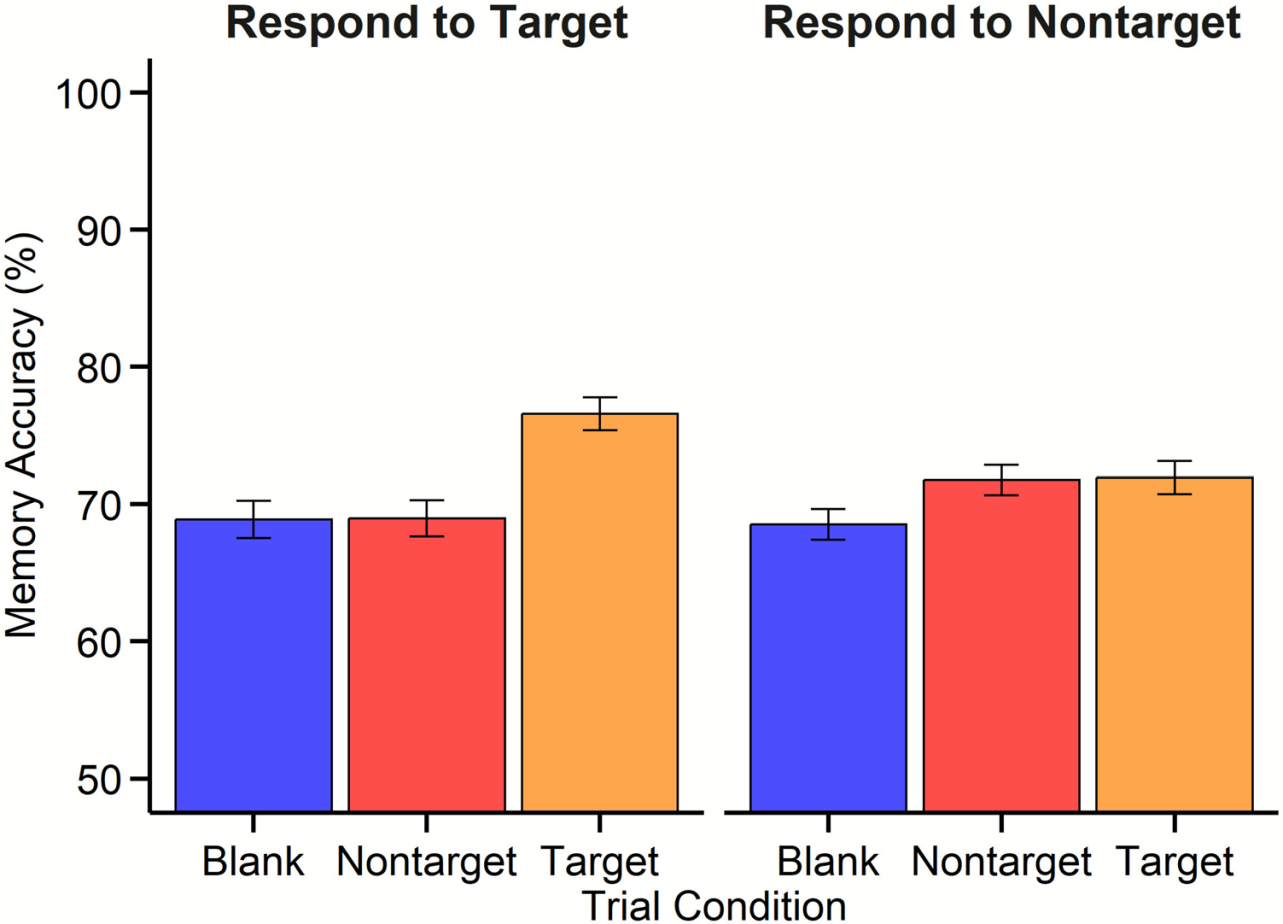
Object memory results in Experiments 1A (left) and 1B (right). Participants responded to target letter trials in Experiment 1A and nontarget letter trials in Experiment 1B. Plotted is the proportion of trials in which participants chose the old object in the 4AFC task. Error bars indicate ±1 within-subject SE of the mean (Morey, 2008). (Color figure online)

Replicating Toh and Lee (2022a), we found an ABE when participants responded to the targets, but not when they responded to the nontargets, reflecting an absolute memory boost (Fig. 2). A generalized linear mixed-effects model (GLMM; binomial link) with fixed effects of trial condition (Blank as reference), response group, and their interaction, and participant-level random intercepts and condition-specific slopes, revealed a significant main effect of trial condition, χ^2^(2) = 19.9, *p* <.001. Recognition was higher for Target than Blank trials (*z* = 2.21, *p* =.027), whereas Nontarget and Blank trials did not differ (*p* =.154). There was no main effect of response group (*p* =.55) and no significant interaction (*p* =.06). Nevertheless, follow-up analyses revealed distinct patterns across response groups.

To assess the role of response demands, separate GLMMs were fit for each group, with trial condition coded using Blank trials as the reference level. This was followed up by planned Target–Nontarget contrasts based on estimated marginal means (Bonferroni-corrected). In the Respond to Target group, trial condition had a significant effect, χ^2^(2) = 17.4, *p* <.001: memory was higher for Target than Blank trials (β = 0.53, *SE* = 0.15, *p* <.001), with no difference between Nontarget and Blank trials (*p* =.78). Estimated marginal means showed highest accuracy for Target-paired images (83%) relative to Nontarget- (74%) and Blank-paired images (74%), and Target-paired images were remembered better than Nontarget-paired images (*z* = 3.74, *p* =.001). In contrast, in the Respond to Nontarget group, trial condition also reached significance, χ^2^(2) = 7.1, *p* =.029, but both Nontarget trials (β = 0.23, *SE* = 0.11, *p* =.046) and Target trials (β = 0.34, *SE =* 0.14, *p* =.015) showed modest memory advantages over Blank trials. Estimated marginal means indicated similar memory accuracy for Nontarget- (76%) and Target-paired images (78%), with  no reliable difference between them (*p* = 1.00), both numerically higher than for Blank-paired images (72%). Together, these results demonstrate a selective Target–Nontarget memory advantage only when responses were made to target.

#### Category and exemplar memory

Because the 4AFC task allows memory to be evaluated at both the category and exemplar levels, we next examined whether the attentional boost extended beyond overall recognition accuracy. Category memory was defined as choosing either the studied object or its within-category foil; exemplar memory was defined as choosing the specific studied object conditional on correct category identification (Sisk & Lee, 2022).

Both measures showed the same qualitative pattern as the overall recognition results. In the Respond to Target group, category and exemplar memory were higher for Target-paired images than for Blank trials, with no difference between Nontarget-paired images and Blank trials. In the Respond to Nontarget group, both category and exemplar memory were numerically higher for Target- and Nontarget-paired images than for Blank trials, but these differences were not statistically significant. Full GLMM results for both category and exemplar memory are provided in Supplements 1 and 2.

Because Spataro et al. (2025) showed that the ABE not only enhances item memory but also increases false alarms to perceptually similar within-category foils in an old/similar/new recognition paradigm, which indicates that perceptual similarity can shape how the ABE influences recognition, we examined whether perceptual similarity predicted exemplar discrimination in our 4AFC memory task. We quantified perceptual similarity using the Learned Perceptual Image Patch Similarity (LPIPS) metric (Zhang et al., 2018), which measures distances in deep neural network feature space calibrated to human perceptual judgments. A GLMM revealed that exemplar memory increased as LPIPS distance increased: participants were more accurate when the studied exemplar and within-category foil were less similar. This similarity effect was independent of trial condition and response group, suggesting that target detection and the need to respond enhance the overall fidelity or accessibility of item representations without selectively modulating sensitivity to perceptual similarity. Full model details are reported in Supplement 3.

## Discussion

Experiment 1 replicated key findings from Toh and Lee (2022a, 2022b), showing that both perceptual and response goals influence the ABE. When these goals aligned, as when participants searched for and responded to the target letter (Experiment 1A), a strong ABE, characterized as the memory enhancement for target-paired images relative to nontarget-paired images, was observed. When goals misaligned, as when participants searched for the target but responded to nontargets (Experiment 1B), the memory difference between target- and nontarget-paired images disappeared.

A novel aspect of Experiment 1 was the inclusion of blank baseline trials. In Experiment 1A, images encoded alone were remembered more poorly than those paired with targets and no better than those paired with nontargets, indicating an absolute boost for target-paired images (Lin et al., 2010; Swallow & Jiang, 2014). In Experiment 1B, memory for both target- and response-paired images were enhanced compared with the blank-paired images.

Together, these results indicate a selective ABE to target-paired images when participants responded to targets and a broader, weaker enhancement to both target- and nontarget-paired images when responding to nontargets. These findings suggest that both target detection and response contribute to the ABE. They provide a basis for comparison with physiological responses measured by pupillometry in Experiment 2. If LC–NE activity drives both the behavioral and physiological effects, we would expect pupil dilation in Experiment 2 to mirror the patterns observed in memory performance here, modulated by target detection and response demands.

## Experiment 2

Pupil size is a well-validated indirect measure of LC–NE activity. Although not a real-time, one-on-one index of LC firing, pupil size correlates with LC firing rates in nonhuman primates and with BOLD activity in the human LC (de Gee et al., 2017; Joshi et al., 2016; Megemont et al., 2022; Murphy et al., 2014; Privitera et al., 2020). Using a go/no-go paradigm, previous studies have shown greater pupil dilation on go trials than on no-go trials (Privitera et al., 2020; Swallow et al., 2019; Yebra et al., 2019). However, because these studies always require responses to targets, they could not disentangle the effects of target detection from response.

Experiment 2 used the same RSVP paradigm as Experiment 1, with targets, nontargets, and blank trials intermixed with equal frequency. In a within-subject design, participants completed two blocks, one in which they responded to the designated target letter (e.g., the letter “E”), and another in which they responded to all other letters. We examined whether changes in response demands modulate pupil dilation across conditions. Background images were omitted to control for luminance variability. As in Experiment 1, we tested whether pupil size would reflect the alignment between perceptual and response goals.

### Method

#### Participants

##### Sample-size determination.

Experiment 2 included 24 participants, predefined based on two previous ABE studies that measured pupil dilation. Cohen’s *d* was 1.24 in Swallow et al. (2019), while Cohen’s *f* was 0.81 in Yebra et al. (2019, Experiment 6). An a priori power analysis using G*Power (Faul et al., 2007) indicated that a sample of 24 participants would provide over .97 power to detect similar effects in a two-sampled test with an alpha of 0.05.

##### Participant characteristics.

All 24 participants were students from the University of Minnesota, who volunteered for extra course credit or monetary compensation. They were fluent in English, had normal or corrected-to-normal vision, were naïve to the study’s purpose, and provided written informed consent. The sample included 14 women and 10 men, with a mean age of 20.8 years (*SD* = 2.6, range: 18–27 years).

#### Equipment

Participants completed the experiment individually in a room with normal interior lighting, with the experimenter present. They maintained a 90-cm viewing distance using a chin rest. Visual stimuli were displayed on a 19-in. CRT monitor (1,024 × 768 pixels, 100-Hz refresh rate). The experiment, programmed in MATLAB using Psychophysics Toolbox (Brainard, 1997), involved monocular eye tracking of the left eye with an EyeLink 1000 eye tracker (SR Research, Mississauga, ON, Canada) at 1000-Hz sampling rate. Eye position was calibrated using a 9-point calibration procedure before the experiment and verified with a drift check every 90 trials.

#### Materials

A medium-gray fixation cross (1.92° × 1.92° visual angle, RGB: 153, 153, 153) was centered on a gray background (RGB: 137, 137, 137) and remained visible throughout the experiment, providing a reference point to help participants maintain fixation. Each trial presented one of three possible conditions: a target letter, a nontarget letter, or a blank screen. The letter set consisted of 20 letters from the Roman alphabet, identical to those used in Experiment 1. Target and nontarget letters were selected like Experiment 1 and were presented in light gray (RGB: 210, 210, 210) at a 0.58° visual angle.

#### Design and procedure

Each participant was pre-assigned one of four possible target letters (“A,” “E,” “H,” or “S”), which remained constant across both task versions (Respond to Target and Respond to Nontarget). Participants were instructed to respond as quickly and accurately as possible. The two response conditions were tested within-subjects, as this design maximizes sensitivity to differences between response conditions and the in-person format allowed for a longer session that accommodated both tasks. All participants completed both task versions in a counterbalanced order: (1) pressing the space bar for the target letter while ignoring nontarget and blank trials (Respond to Target), and (2) pressing the space bar for any of the 19 nontarget letters while ignoring the target and blank trials (Respond to Nontarget). Participants completed 18 practice trials before each task.

Each task consisted of three blocks of 90 trials, with a drift check preceding each block. On each trial, participants fixated on a central point and pressed the space bar to begin. If the eye tracker confirmed central fixation, the RSVP stream appeared; recalibration was conducted as needed. Participants were instructed to maintain steady fixation while blinking naturally but minimizing excessive blinking. Every 18 trials (five times per block), the fixation cross thickened, signaling a brief 3-s rest. At the end of each block, participants received accuracy feedback. If accuracy fell below 90%, they were reminded to strive for 100% accuracy while maintaining quick responses.

#### Pupil data processing

Pupil area was recorded using an EyeLink 1000 eye tracker. EDF files were converted into MATLAB structures using the *Edf2Mat* toolbox (Kovach, 2011). For each participant, raw data were segmented into trials using predefined event markers to align pupil data with stimulus onset and responses. Data were then cleaned and processed in MATLAB following Riley et al. (2024). Blink artifacts were corrected via linear interpolation, with a 50-ms margin applied before and after each blink. Rapid fluctuations in pupil size were identified using dilation speed derivatives, with extreme values exceeding eight times the median absolute deviation flagged and interpolated (Kret & Sjak-Shie, 2019). The cleaned signal was smoothed using rLOESS, and baseline normalization was performed by subtracting the mean pupil size during the 200-ms preceding stimulus onset from each trial. Outliers beyond the 1 st and 99th percentiles for each participant were removed.

### Results

#### Letter detection

##### Respond to target.

When participants were asked to respond to target letters, they responded to the target letters 98.9% (*SE* = 0.6%) of the time, with a mean RT of 598 ms (*SE* = 17 ms). They false alarmed to nontarget letters 1.3% (*SE* = 0.3%) of the time and to the blank trials 0.3% (*SE* = 0.1%) of the time. The false-alarm rate for nontarget letters was significantly higher than the false-alarm rate for blank trials, *t*(23) = 3.09, *p* =.005, Cohen’s *d* = 0.63, suggesting that it was more difficult for participants to withhold responses to the nontarget letters compared with the blank trials, consistent with Experiment 1.

##### Respond to nontarget.

Consistent with Experiment 1, participants responded to nontarget letters with high accuracy (98.9%, *SE* = 0.3%), but with a slower mean RT of 659 ms (*SE* = 19 ms) compared with when they responded to targets. False-alarm rates to target letters (4.7%, *SE* = 0.9%) were significantly higher than to baseline blanks (0.2%, *SE* = 0.1%), *t*(23) = 5.19, *p* <.001, Cohen’s *d* = 1.06, indicating that blank trials imposed lower attentional demands than target letters.

##### Combined analysis of trial condition and response demands.

When hit and false-alarm rates were combined into a *d’* measure, performance in Respond to Target (*M* = 4.81, *SE* = 0.09) was significantly higher than in Respond to Nontarget (*M* = 4.44, *SE* = 0.11), *t*(23) = 2.97, *p* =.007, Cohen’s *d* = 0.61. Mean RT was also significantly faster in Respond to Target than in Respond to Nontarget, *t*(23) = 3.64, *p* =.001, Cohen’s *d* = 0.74. These results replicate the pattern from Experiment 1: the task was easier when the target letter aligned with the key press response, compared with when the response required withholding for targets.

#### Pupillary time course

##### **Respond to target**. 

**Fig. 3:**
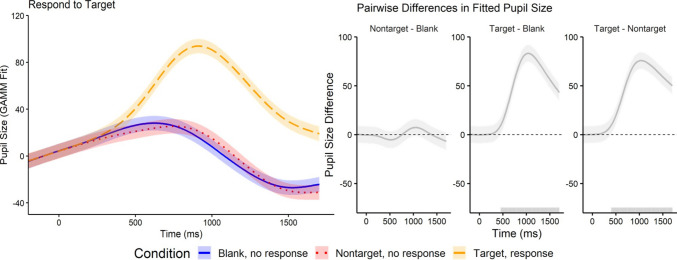
Left panel: Generalized additive mixed model (GAMM) fit of pupil size by trial condition when participants respond to Target letters. The plot illustrates the predicted pupil size across time for each trial condition, with shaded regions representing 95% confidence intervals. The “Blank, no response” condition is represented by the solid blue line, “Nontarget, response” by the dotted red line, and “Target, no response” by the long-dashed orange line. Time 0 ms corresponds to stimulus onset. Right panel: Pairwise differences in the GAMM-fitted pupil size after FDR correction between trial conditions over time. Solid gray lines indicate estimated differences. Gray shading along the *x*-axis marks time windows where the differences reached statistical significance. These significant intervals were determined using FDR correction for multiple comparisons between pairwise condition differences. (Color figure online)

We performed a generalized additive mixed model (GAMM) analysis to examine pupillary responses across different trial types (Blank, Nontarget, and Target). The GAMM modeled pupil size as a smooth function of time, with separate smooths for each condition, and included random intercepts for participants to account for repeated measures. Time was normalized prior to modeling, and the model was fit using the *gamm4* package in R. We verified that residuals were approximately normal and homoscedastic, and there was minimal autocorrelation, supporting the validity of the model assumptions. Figure 3 (left panel) shows the pupil size time courses predicted by the GAMM. At baseline (prior to stimulus onset), pupil size did not differ significantly across trial conditions. Following stimulus onset (time = 0), pupil size increased sharply and then decreased. However, this decline was attenuated during Target trials, where participants detected a target and made a response. Pupil size remained larger throughout Target trials (*M* = 43.05) compared with Nontarget (*M* = 3.67) and Blank (*M* = 3.81) trials.

Figure 3 (right panel) shows the pairwise differences in pupil size, revealing distinct temporal patterns of dilation across conditions. To quantify these patterns, we conducted pairwise comparisons with False Discovery Rate (FDR) correction in R. The most robust effect was observed for Target trials: pupil size significantly diverged from Blank trials starting at 455 s after stimulus onset and remained significantly different through the end of the trial (mean Cohen’s *d* = 1.53, maximum *d* = 3.24). We also found a significant difference between Nontarget and Target trials, starting at 404ms and lasting throughout the trial (mean Cohen’s *d* = 1.53, maximum *d* = 2.94). Pupillary responses during Nontarget and Blank trials did not differ significantly from each other throughout the trial (mean Cohen’s *d* = 0.17, maximum *d* = 0.39). These results suggest that detecting and responding to a target evoke a significant pupillary response compared with ignoring nontarget letters or blank trials.

Because the response conditions were tested between subjects in Experiment 1 but within subjects in Experiment 2, we examined whether block order in Experiment 2 (Respond to Target first vs. Respond to Nontarget first; *N* = 12 per group) influenced the pupillary results. Response order affected the magnitude of pupil dilation but did not change the pattern of condition effects (Supplement Fig. 4A). Target trials elicited a substantially larger dilation than blank trials (β = 48.04, *S*E = 0.34, *t* = 139.38, *p* <.001), whereas nontargets did not differ from blanks (β = 0.09, *SE* = 0.34, *t* = 0.25, *p* =.80). Participants who completed the Respond to Nontarget block first showed a reduced overall target-evoked response (interaction β = –17.61, *SE* = 0.49, *t* = –36.12, *p* <.001), but this amplitude shift did not alter the qualitative pattern: nontargets remained statistically indistinguishable from blanks, and this relationship did not vary by response order (nontarget × order β = –0.44, *SE* = 0.49, *t* = –0.91, *p* =.36). Thus, in both groups, nontarget and blank trials produced similarly low dilation levels, whereas targets continued to evoke markedly larger responses, preserving the qualitative pattern of target > nontarget = blank.

**Fig. 4:**
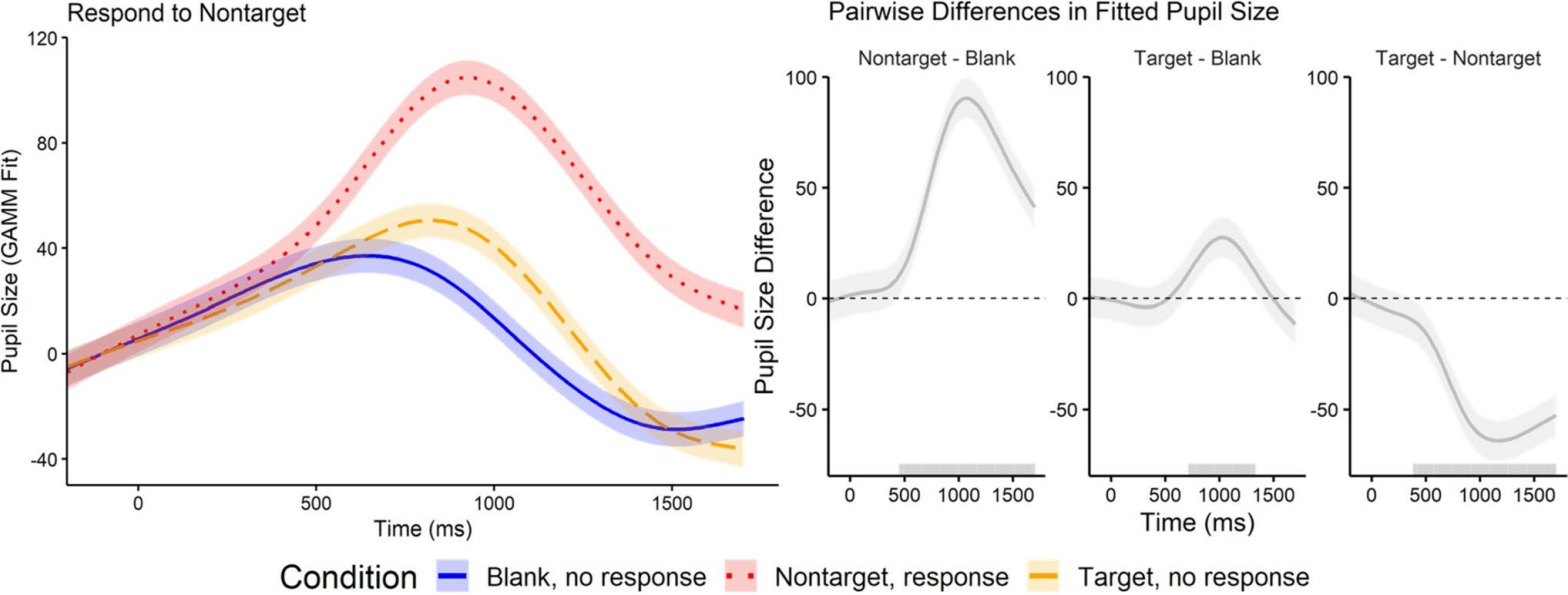
Left panel: Generalized additive mixed model (GAMM) fit of pupil size by trial condition when participants respond to Nontarget letters. The plot illustrates the predicted pupil size across time for each trial condition, with shaded regions representing 95% confidence intervals. The “Blank, no response” condition is represented by the solid blue line, “Nontarget, response” by the dotted red line, and “Target, no response” by the long-dashed orange line. Time 0 ms corresponds to stimulus onset. Right panel: Pairwise differences in the GAMM-fitted pupil size after FDR correction between trial conditions over time. Solid gray lines indicate estimated differences. Gray shading along the x-axis marks time windows where the differences reached statistical significance. These significant intervals were determined using FDR correction for multiple comparisons between pairwise condition differences. (Color figure online)

##### Respond to nontarget.

When participants responded to Nontarget trials, there were also no significant differences in starting pupil size between trial condition. Following stimulus onset, pupil size rapidly increased before decreasing (Fig. 4, left panel). Pupil size during the Nontarget trials that were responded showed the most pronounced increase (*M*= 48.7). Crucially, although participants did not respond to Target trials, pupillary responses during Target trials were elevated (*M* = 13.9) compared with Blank trials (*M* = 7.3).

Figure 4 (right panel) shows the pairwise differences (FDR corrected) in GAMM-fitted pupil size. The most robust effect was observed in Nontarget trials that were responded, which significantly diverged from Blank trials starting at 455 ms after stimulus onset and remained significant throughout the trial (mean Cohen’s *d* = 1.43, maximum *d* = 3.12). A significant difference was also found between Nontarget and Target trials, beginning at 385 ms and lasting throughout the trial (mean Cohen’s *d* = 1.14, maximum *d* = 2.10). Importantly, although participants did not respond to Target trials, a significant difference between Target and Blank trials was observed from 716 ms (around the time of peak dilation) to 1,333 ms after stimulus onset (mean Cohen’s *d* = 0.38, maximum *d* = 1.12).

These results suggest that response requirements play a major role in modulating phasic pupillary responses. However, response alone cannot fully explain the observed effects. If response were the only factor, we would not have observed significant differences between Target and Blank trials. This pattern is distinct from when participants responded to Target trials. When participants responded to Target trials, Nontarget and Blank trials, neither of which received a response, did not differ significantly from each other.

Like before, response order affected the overall magnitude of dilation but did not change the qualitative pattern of condition effects (Supplement Fig. 4B). Nontarget trials, which required a behavioral response in this block, produced substantially larger dilation than blank trials (β = 40.41, *SE* = 0.36, *t* = 111.43, *p* <.001), and this effect remained positive in both response-order groups (interaction β = 2.08, *SE* = 0.51, *t* = 4.06, *p* <.001). Targets, although behaviorally irrelevant, also elicited significantly larger dilation than blanks (β = 1.75, *SE* = 0.36, *t* = 4.83, *p* <.001), and this difference was even greater for participants who completed the Respond to Nontarget block first (interaction β = 9.79, *SE* = 0.51, *t* = 19.09, *p* <.001). Thus, in both response-order groups, pupil dilation followed the same qualitative pattern: nontarget > target > blank.

##### Combined analysis of trial condition and response demands.

To jointly test the contributions of stimulus identity and response requirements, we fit a unified GAMM with trial condition (Blank, Nontarget, Target) and response requirement (go, no-go), condition-specific smooths, and subject-specific random smooths. The model explained 79% of the variance in pupil size. There were robust main effects of trial condition and response requirement and a strong Trial Condition × Response Requirement interaction (all *p*s < 2×10⁻^16^). Go trials produced the largest dilations, and trial condition exerted the greatest influence when a response was required. Crucially, Target–no-go trials produced greater dilation than Blank–no-go trials, confirming that stimulus identity modulates pupil dilation independently of motor demands.

#### Memory-pupil index comparison

To directly compare the magnitude of the boost across Experiments 1 and 2, we computed parallel indices for memory and pupillometry. For each participant, we calculated a relative boost index (Target − Nontarget for Respond to Target; Nontarget − Target for Respond to Nontarget) and an absolute boost index (Target − Blank or Nontarget − Blank), and baseline-normalized each value by dividing by that participant’s Blank-condition mean.

Normalized indices in the Memory experiment were small (means = 0.005–0.18), whereas the corresponding Pupil indices showed substantially greater variability but remained comparable in overall magnitude (means = –2.49 to 7.73; see Supplement 5 for Mean ± *SE* of all normalized indices). Independent-samples *t* tests revealed no significant differences between the memory and pupillary experiments for any of the four normalized boost measure (all *t*s < 1.0, all *p*s >.38), with uniformly small effect sizes (|*d*| = 0.04–0.29). Across all indices, Bayesian independent-samples *t* test also favored the null (BF_01_ = 2.27–3.71), indicating anecdotal-to-moderate evidence that the boost magnitudes derived from pupil dilation and memory performance are statistically indistinguishable.

### Discussion

Experiment 2 used pupil dilation as an index of LC-NE activity to test how target detection and response demands shape phasic arousal. Across both response conditions, pupil dilation reflected contributions from both processes, but their relative influence depended on the task. When participants responded to targets, pupil dilation was substantially greater on target trials than on nontarget and blank trials, which did not differ. In contrast, when participants responded to nontargets, Target–no-go trials still elicited greater dilation than Blank-no-go trials, indicating that target detection modulated pupil size even without the need for behavioral response. These effects did not change as a result of response order, confirming that they reflected trial-level cognitive demands.

This pattern partially aligned with the behavioral results from Experiment 1. When participants responded to targets, both pupil dilation and memory were enhanced for target-paired images. However, when they responded to nontargets, the two measures diverged: memory accuracy showed a modest boost for both target- and nontarget-paired images relative to blank-paired images; pupil dilation varied, showing a strong response to nontargets and a moderate response to targets. Despite these qualitative differences, both memory and pupillary measures trended in the same direction (nontarget and target > blank) and the normalized boost indices from the two experiments were statistically indistinguishable.

While both behavioral (memory) and physiological (pupil dilation) measures were influenced by target detection and response demands, pupil dilation appeared more strongly driven by response. This suggests that although pupil dilation can reflect behavioral effects, the underlying mechanisms are unlikely the same.

## General discussion

The attentional boost effect, a well-established but intriguing phenomenon, demonstrates that detecting and responding to attention-demanding targets surprisingly improves the processing of concurrent, unrelated stimuli. While evidence suggests a role for phasic LC activity in mediating a broad neural boost after target detection (Swallow et al., 2012, 2019; Yebra et al., 2019), the precise drivers of this effect remain unclear. Here, we show that both target detection and response contribute to the ABE, as reflected in memory for concurrently presented stimuli and in pupil dilation. However, behavioral and physiological responses do not align perfectly: pupil dilation is more sensitive to response demands than are behavioral measures. These findings support the role of the LC–NE system in both drivers of the ABE—target detection and response—while also suggesting that the relationships among LC firing, pupil dilation, and the behavioral ABE are not one-to-one.

In Experiment 1, participants monitored an RSVP stream consisting of letters and blank squares while simultaneously encoding background images. They were randomly assigned to one of two groups: one group responded to a prespecified target letter (Respond to Target), while the other group responded to any letter except the target (Respond to Nontarget). The use of a large and varied set of nontarget letters ensured that the most efficient strategy in both groups was to search for the designated target letter and then decide whether to respond based on the task instructions. This design effectively dissociated perceptual target detection from the requirement to respond.

We found a robust ABE in the Respond to Target group: memory for images paired with target letters was significantly better than memory for images paired with nontarget letters and blank baseline trials. This reflects not just a relative but an absolute memory benefit for target-paired images. In contrast, in the Respond to Nontarget group, memory performance for images paired with targets and nontargets trials received modest enhancement relative to blank trials. These results demonstrate that the need to respond also contributes to the ABE, challenging a single-locus account that attributes it solely to target detection or response.

Although response inhibition has been proposed as a mechanism underlying the ABE, evidence is mixed. Increasing inhibitory load does not reliably modulate memory (Yebra et al., 2019). Moreover, while Makovski et al. (2013) reported enhanced memory for infrequent no-go trials ( suggesting that stronger inhibition did not impair memory), that study required direct responses to the memory items, deviating from the standard ABE paradigm. In the present study, we showed that memory performance also did not scale with response inhibition in an ABE paradigm. When participants responded to target letters, memory for nontarget-paired images (no-go trials) did not differ from blank-paired images despite higher inhibitory demands, as indicated by elevated RSVP false-alarm rates. When participants responded to nontarget letters, memory for target-paired images (no-go trials) was not reduced relative to blank-paired images and was comparable to memory for nontarget-paired images (go trials). Together, these findings indicate that response inhibition does not account for memory performance in this task; instead, the absence of an ABE in the Respond to Nontarget group is more likely due to a misalignment between perceptual and response goals.

Our findings also align with work showing that the ABE selectively strengthens exemplar-level representations (Sisk & Lee, 2022). This effect was not modulated by the perceptual similarity of within-category foils: similarity predicted overall exemplar accuracy but did not interact with trial condition. This contrasts with Spataro et al. (2025), which showed greater false alarms for similar foils after target-paired encoding. The discrepancy likely reflects task differences: 4AFC minimizes global familiarity inflation, whereas old/similar/new tasks amplify it. This suggests that the ABE enhances item-specific memory, with related-familiarity effects emerging only under tasks that probe absolute familiarity.

In Experiment 2, we examined pupil dilation as an index of LC–NE activity. If the LC–NE mechanism underlies the ABE, then much like behavioral measures, phasic pupillary responses should be modulated by both perceptual and response goals. Our findings support this hypothesis: when participants responded to targets, they exhibited the largest pupil dilation, while nontarget and blank trials evoked comparable responses. The fact that nontarget trials required greater cognitive control and inhibition than blank trials, as indicated by higher false-alarm rates in the RSVP task, suggests that task-related effort alone does not explain the pupillary data. In contrast, when participants responded to nontargets, the greatest pupil dilation occurred on nontarget trials requiring a response. Importantly, pupil dilation during target letter trials (despite not requiring a response) exceeded baseline. Thus, both target detection and response contributed to pupil dilation.

Although the overall patterns were qualitatively similar, pupil dilation and memory measures did not align perfectly. In the Respond to Nontarget condition, nontarget trials produced large pupil dilations in Experiment 2, yet in Experiment 1 they yielded only modest memory enhancement relative to blank trials and did not differ significantly from target trials. This divergence suggests that the relationship among LC–NE activity, pupil dilation, and the behavioral ABE is more complex than a simple direct correspondence.

Several factors may have contributed to the divergence between behavioral and physiological measures. First, pupil dilation may be influenced by neural systems beyond the LC–NE system, including other brain regions and neuromodulatory pathways (Joshi et al., 2016; Reimer et al., 2016; Wang & Munoz, 2015). If the behavioral ABE primarily originates from the LC–NE system, but pupil dilation is influenced by additional factors, the two measures would only partially align. Recent development in neuroimaging now allows more direct measurement of activity in the LC (Turker et al., 2021). Future work could leverage these techniques to simultaneously record behavior, pupil dilation, and LC activity, providing a more direct assessment of how target detection and response affect the three ABE indices.

Second, as an immediate physiological response, pupil dilation might be more sensitive to certain aspects of motor execution like pressing a key whereas the behavioral ABE, a measure of concurrent memory, may be less affected by the motor act itself. To clarify the relationship between perceptual and response targets and pupil dilation, future studies could replace the overt button press with a covert response, such as silent counting. This could help determine if motor execution is a key contributor to observed pupil dilation, or if pupillary responses more broadly reflect decision-making related to response preparation, irrespective of overt motor output.

Finally, the two experiments imposed different task demands: participants in Experiment 1 performed a dual task of letter detection and image encoding, whereas those in Experiment 2 only monitored for letters without concurrent image encoding. Intuitively, one might expect that if the pupil responds strongly to specific letter events (e.g., nontargets requiring a response) in the absence of background images, it should continue to do so when images are added. However, the increased cognitive load of the dual-task in Experiment 1 could modulate the LC–NE dynamics or its downstream effects. Because pupil diameter is not a one-to-one readout of LC activity and is also sensitive to factors such as attentional load (Alnæs et al., 2014; Wahn et al., 2016) and attentional capture (Marois & Vachon, 2018), whether the same pupillary signatures would arise under dual-task conditions remains an open empirical question for future work.

**Fig. 5:**
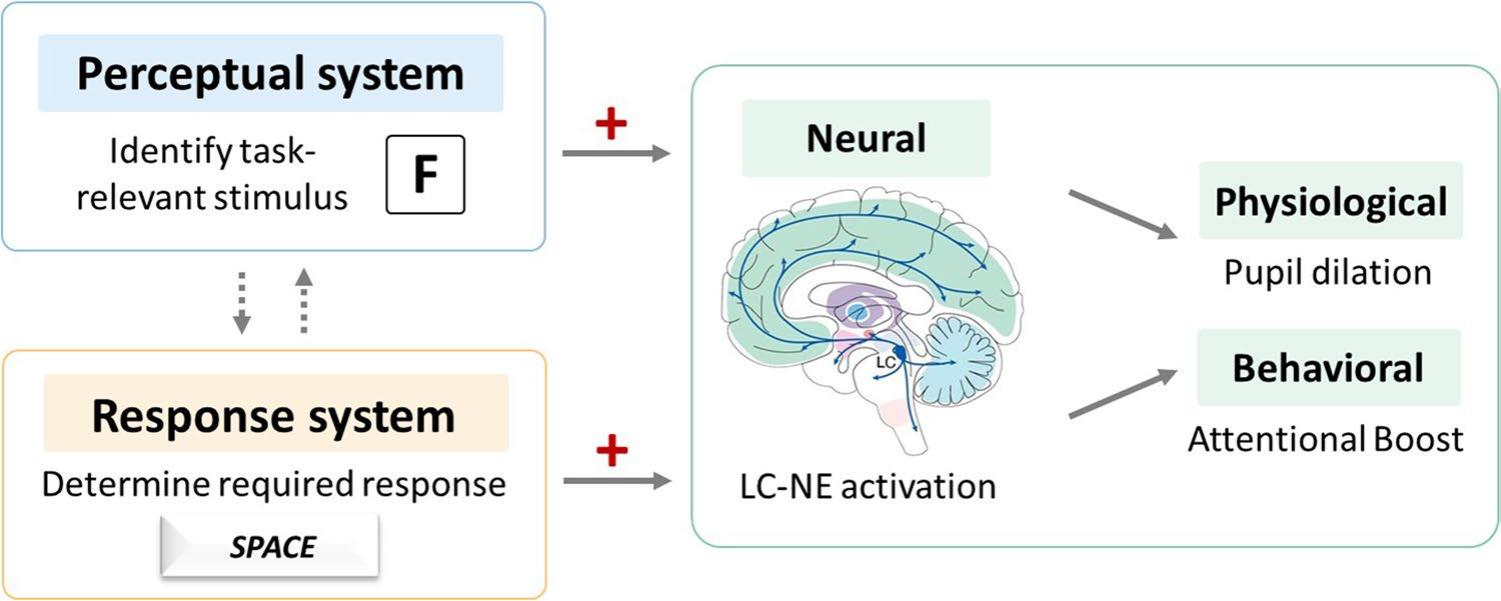
Schematic illustration of how target detection and the need to respond jointly contribute to phasic locus coeruleus-norepinephrine (LC–NE) activation. The perceptual system identifies task-relevant stimuli (e.g., a target letter), while the response system determines whether a behavioral response is required (e.g., pressing the space bar). The LC–NE system is most strongly activated when both perceptual and response goals are engaged. This co-activation produces the largest pupil dilation and is associated with the attentional boost effect (ABE). In contrast, when only one component is engaged (e.g., perceptual detection without response, or response without perceptual tagging as a target), LC–NE activation is weaker, and the ABE may not be detectable. The image of Locus Coeruleus in this figure was adapted from Fig. 1 of Breton-Provencher et al. (2021), published by *Frontiers in Neural Circuits* under the Creative Commons Attribution License. (Color figure online)

Despite these nuances, this study demonstrated that both target detection and the need to respond contribute to the ABE, evident in both behavioral and pupillary measures. These two factors can be conceptualized as a dual-input system, akin to light controlled by two dimmer switches: one for perceptual detection and one for response demand (Fig. 5). Engaging either component alone brightens the signal modestly, but when both are active, they strongly drive the LC–NE system, resulting in enhanced memory for concurrent stimuli. These findings align with models proposing that the LC–NE system functions as a gating mechanism for adaptive gain, selectively enhancing processing when behaviorally relevant events align with task goals and action plans (Aston-Jones & Cohen, 2005). In this view, perceptual detection alone may initiate a partial LC response, but full engagement and the associated cognitive benefits may require coordinated activation of both perceptual and response systems.

## Conclusion

In a world of information overload, what draws our attention? Some moments pass unnoticed, while others are deeply processed and remembered. While internal factors like motivation and goals are known to shape attention, our findings show that externally imposed task goals, specifically those requiring both target detection (perceptual goal) and a behavioral response (response goal), can effectively simulate a similar attentional enhancement. These effects are reflected in both memory performance and pupil dilation, though the latter is more sensitive to response. Understanding the drivers of the attentional boost effect may inform technologies like augmented reality, where well-timed prompts could improve attention, learning, and task performance.

## Supplementary Information

Below is the link to the electronic supplementary material.

Supplementary materials (DOCX 272 KB)

## Data Availability

These experiments were not pre-registered. De-identified data in an aggregated format are available on the Open Science Framework (https://osf.io/7a8v6/).

## Code availability

The experimental scripts and demonstration videos of both experiments are available on the Open Science Framework (https://osf.io/7a8v6/).

## Declarations

### Open practices statement

These experiments were not preregistered. De-identified data in an aggregated format, experimental scripts, and demonstration videos of both experiments are available on the Open Science Framework (https://osf.io/7a8v6/).
