# Supplementary material for: Roles of target detection and behavioral response on pupil dilation and concurrent memory: An attentional boost study
Source: Atten Percept Psychophys. 2026 Feb 23;88(3):80. doi: 10.3758/s13414-025-03221-4 (PMC12929347; doi:10.3758/s13414-025-03221-4)
Supplement: Supplementary file 1 — Supplementary materials (DOCX 272 KB) [file 13414_2025_3221_MOESM1_ESM.docx]

**Supplementary Materials**

**Supplement 1: Category Memory**

A generalized linear mixed-effects model (GLMM; binomial link) examined category memory accuracy as a function of trial condition (Blank, Nontarget, Target) and response group (Respond to Target vs. Respond to Nontarget), with random intercepts and slopes for condition by participant. The model revealed a significant main effect of trial condition, χ²(2) = 11.4, *p* = .003, indicating that category memory performance varied across trial types. The main effect of response group was not significant, χ²(1) = 0.04, *p* = .84, and the interaction between trial condition and response group showed a marginal trend, χ²(2) = 5.15, *p* = .076.

To assess the role of response demands, separate GLMMs were fit for each group, followed by planned pairwise contrasts based on estimated marginal means (Bonferroni-corrected). In the Respond to Target group, trial condition had a significant effect on accuracy, χ²(2) = 11.8, *p* = .003. Planned contrasts showed that accuracy was higher for Target trials than for Blank trials (odds ratio = 0.56, Bonferroni-corrected *p* = .019) and Nontarget trials (odds ratio = 0.52, Bonferroni-corrected *p* = .002), whereas Blank and Nontarget trials did not differ (odds ratio = 1.08, *p* = 1.00). Estimated marginal means indicated the highest accuracy for Target-paired images (93.7%), relative to Nontarget- (88.4%) and Blank-paired images (89.1%). In contrast, in the Respond to Nontarget group, trial condition did not significantly affect accuracy, χ²(2) = 1.36, *p* = .51. Estimated marginal means were comparable across Filler (88.4%), Nontarget (88.9%), and Target trials (89.8%), and no Bonferroni-corrected pairwise contrasts reached significance (all odds ratios between 0.86 and 0.95, all *ps* ≥ .74).


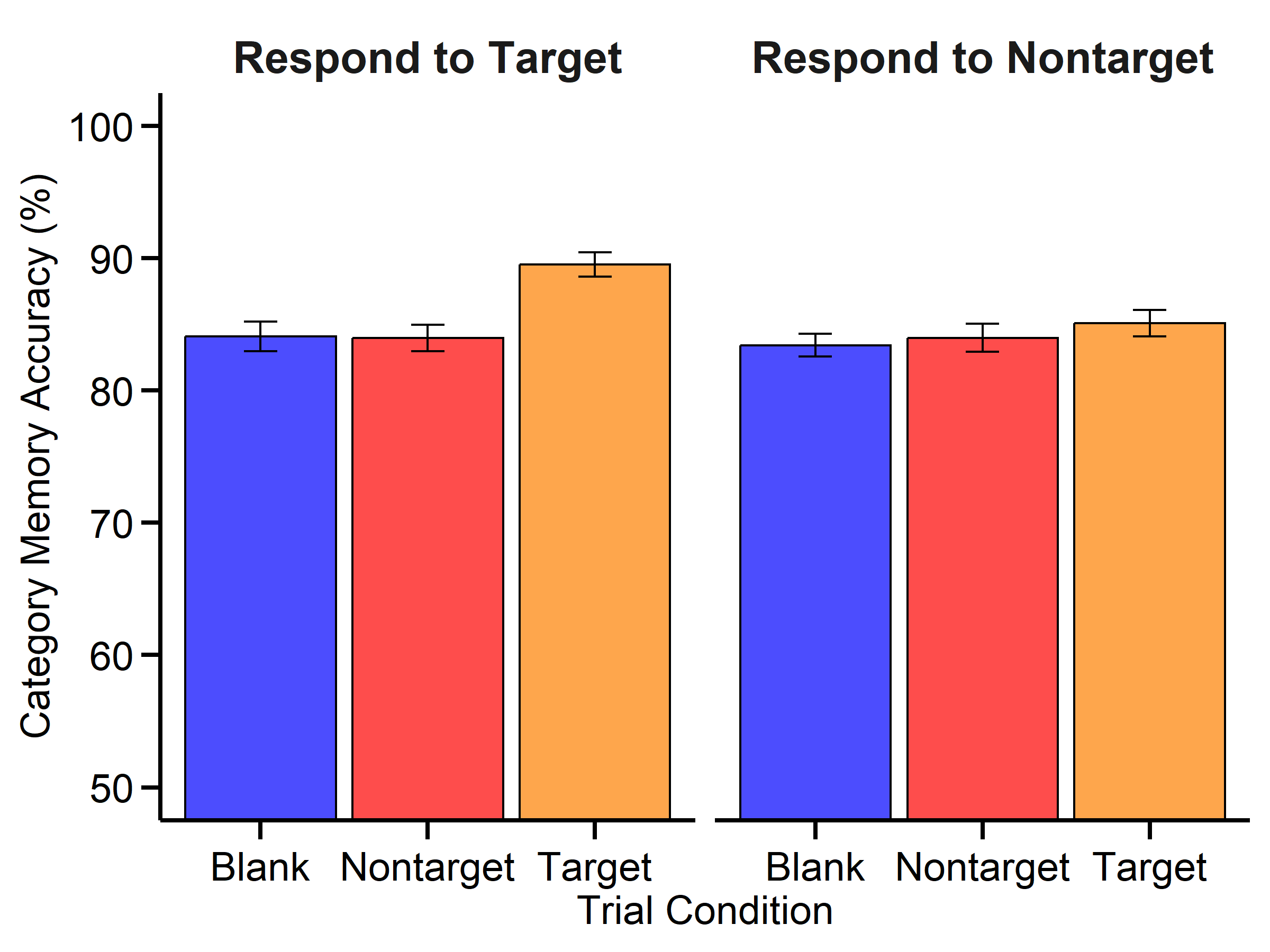


***Supplementary Figure 1.*** *Category memory accuracy in Experiments 1A (left) and 1B (right). Participants responded to target letter trials in Experiment 1A and nontarget letter trials in Experiment 1B. Plotted is the proportion of trials in which participants chose the category of the old object in the 4AFC task. Error bars indicate ± 1 within-subject S.E. of the mean.*

**Supplement 2: Exemplar Memory**

To assess exemplar-level memory performance, we computed accuracy conditional on correct category identification, i.e., P (correct exemplar ∣ correct category). This means we measured how often participants chose the correct exemplar only on trials where they had already chosen the correct category. This approach controls for category-level errors, so the measure reflects memory for specific exemplars rather than general category recognition. A GLMM (binomial link) examined exemplar memory accuracy as a function of trial condition (Blank, Nontarget, Target) and response group (Respond to Target vs. Respond to Nontarget), with random intercepts and slopes for condition by participant. The model revealed a significant main effect of trial condition, χ²(2) = 10.3, *p* = .006, indicating that exemplar memory varied across trial types. The main effect of response group was not significant, χ²(1) = 0.71, *p* = .40, and the interaction between trial condition and response group was also nonsignificant, χ²(2) = 2.41, *p* = .30.

To assess the role of response demands on exemplar memory, separate GLMMs were fit for each group, followed by planned pairwise contrasts based on estimated marginal means (Bonferroni-corrected). In the Respond to Target group, trial condition had a significant effect on exemplar memory, χ²(2) = 8.32, *p* = .016. Planned contrasts indicated that exemplar memory was higher for Target trials than for Blank trials (odds ratio = 0.66, Bonferroni-corrected *p* = .046), with a similar trend relative to Nontarget trials that did not survive correction (odds ratio = 0.65, *p* = .054). Blank and Nontarget trials did not differ (odds ratio = 1.01, *p* = 1.00). Estimated marginal means showed the highest exemplar memory for Target-paired images (88.8%), relative to Nontarget- (83.9%) and Blank-paired images (84.0%). In contrast, in the Respond to Nontarget group, trial condition did not significantly affect exemplar memory, χ²(2) = 3.25, *p* = .20. Estimated marginal means were comparable across Blank (83.7%), Nontarget (86.6%), and Target trials (86.1%), and no Bonferroni-corrected pairwise contrasts reached significance (all odds ratios between 0.80 and 1.04, all *ps* ≥ .27).


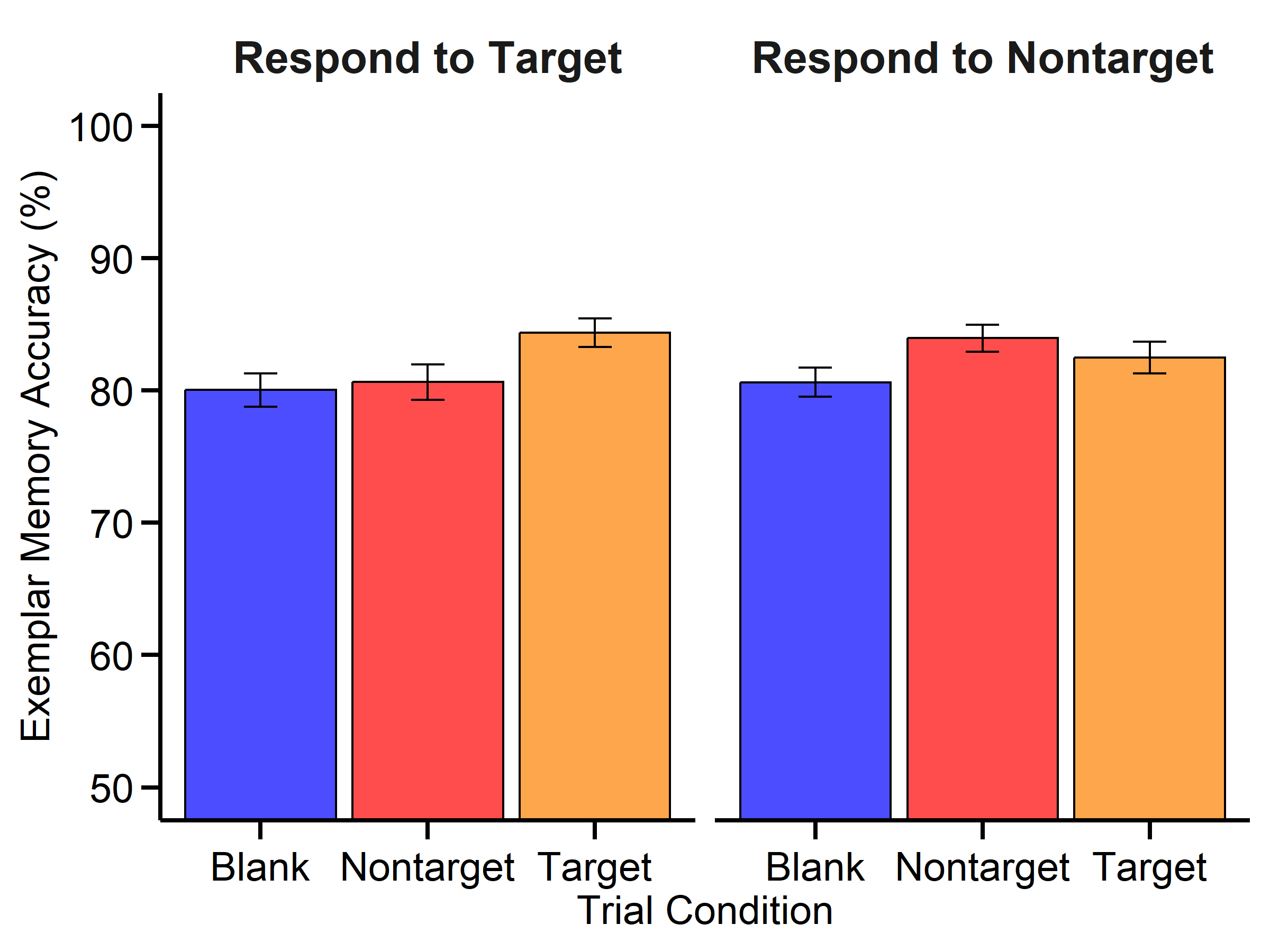


***Supplementary Figure 2.*** *Exemplar memory accuracy in Experiments 1A (left) and 1B (right). Participants responded to target letter trials in Experiment 1A and to nontarget letter trials in Experiment 1B. Plotted is the proportion of trials in which participants correctly identified the exemplar of the old object, considering only trials in which the correct category was selected in the 4AFC task. Error bars indicate ± 1 within-subject SEM.*

**Supplement 3: Effect of Within-Category Foil Similarity on Exemplar Memory**

Perceptual similarity was quantified using the Learned Perceptual Image Patch Similarity (LPIPS) metric (Zhang et al., 2018), which measures the distance between images based on differences in deep neural network feature representations, with feature weights calibrated to human perceptual judgments. Each exemplar pair within a category used in Experiment 1 (i.e., the encoded item and its corresponding within-category foil) was processed through a pretrained LPIPS model implemented in PyTorch, producing a continuous distance score (lower = more perceptually similar). These LPIPS distances were then *z*-scored across all items before being entered into the model.

To examine whether perceptual similarity among within-category foils influenced exemplar recognition performance, we fit a GLMM (binomial link) predicting exemplar memory accuracy from *z*-scored LPIPS distance, response group (Respond to Target vs. Respond to Nontarget), and test condition (Target, Nontarget, or Blank). Random intercepts were included for both participant and item category.

The model revealed significant main effects of perceptual similarity, χ²(1) = 9.66, *p* = .002, and trial condition, χ²(2) = 10.95, *p* = .004, but no main effect of response group, χ²(1) = 0.01, *p* = .94. No higher-order interactions reached significance (all *ps* > .13). As shown in Figure S3, exemplar memory accuracy increased as LPIPS distance increased: participants were more accurate when the studied and foil images were less perceptually similar. Thus, perceptual distinctiveness between exemplars predicted better recognition, confirming that within-category confusions were driven by visual similarity.

Follow-up analyses conducted separately for the two response groups yielded the same pattern. For participants who responded to target trials, exemplar memory accuracy showed main effects of LPIPS distance, χ²(1) = 10.82, *p* = .001, and trial condition, χ²(2) = 10.78, *p* = .005, but no interaction between them, χ²(2) = 1.34, *p* = .51. For those who responded to nontarget trials, there was again a main effect of LPIPS distance, χ²(1) = 4.95, *p* = .026, but neither the main effect of trial condition, χ²(2) = 3.95, *p* = .14, nor the interaction, χ²(2) = 0.12, *p* = .94, was significant.

Together, these results indicate that exemplar memory declines as perceptual similarity increases, regardless of trial condition or response group. The absence of interactions suggests that the attentional boost effect enhances the overall fidelity or accessibility of item representations without selectively modulating sensitivity to perceptual similarity.


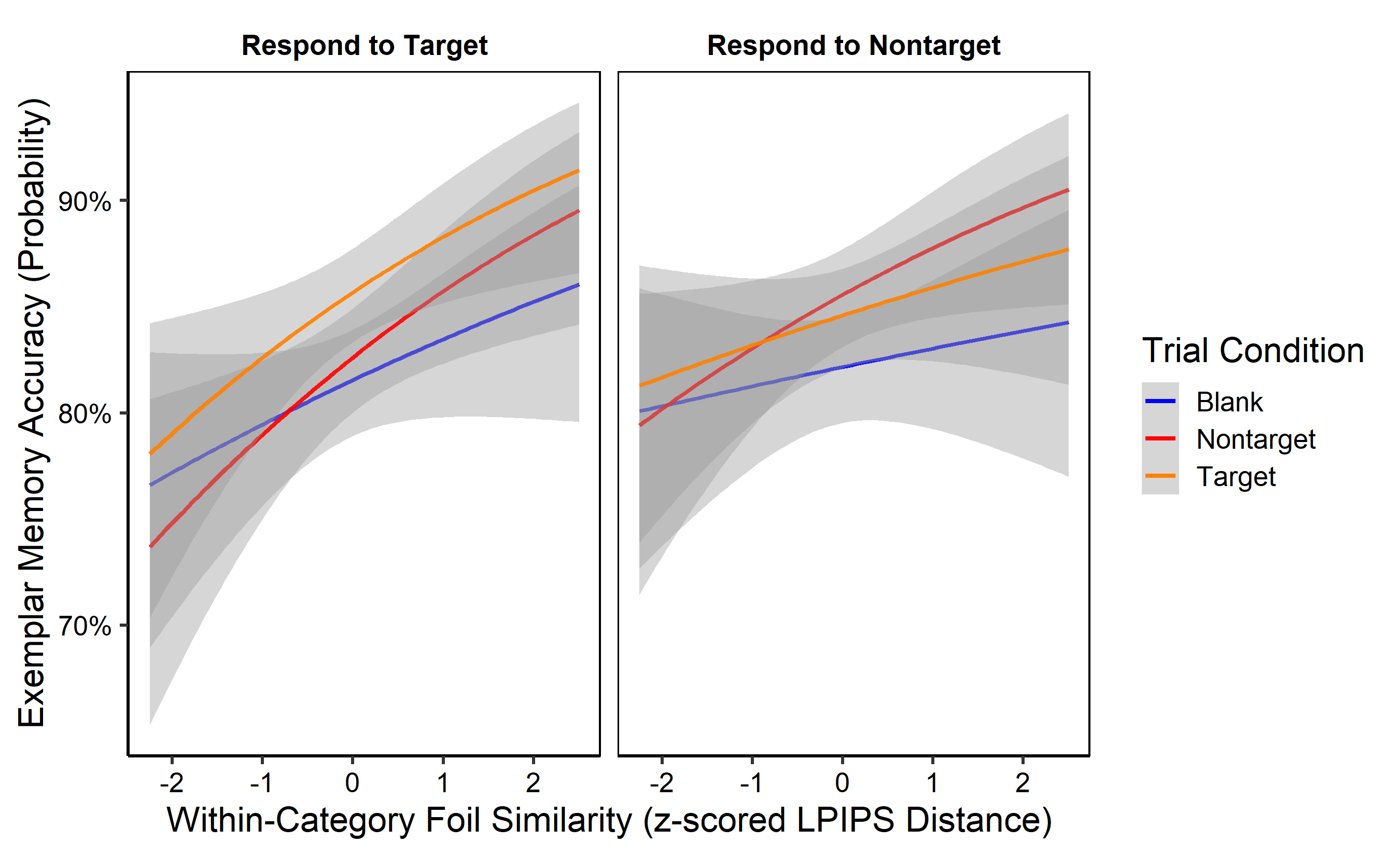


***Supplementary Figure 3.*** *Exemplar memory accuracy as a function of within-category foil similarity. Plotted is the probability of correctly identifying the studied exemplar (conditional on correct category identification) as a function of the z-scored LPIPS distance between the encoded object and its within-category foil. Higher values on the x-axis indicate that the foil was more visually dissimilar from the studied exemplar. Logistic regression curves show the predicted probability of correct exemplar memory for each trial condition (Blank, Nontarget, Target). Shaded bands indicate 95% confidence intervals. Panels separate the two response groups (Respond to Target vs. Respond to Nontarget).*

**Supplement 4: Effect of Response Order on Pupillary Time Course**

**
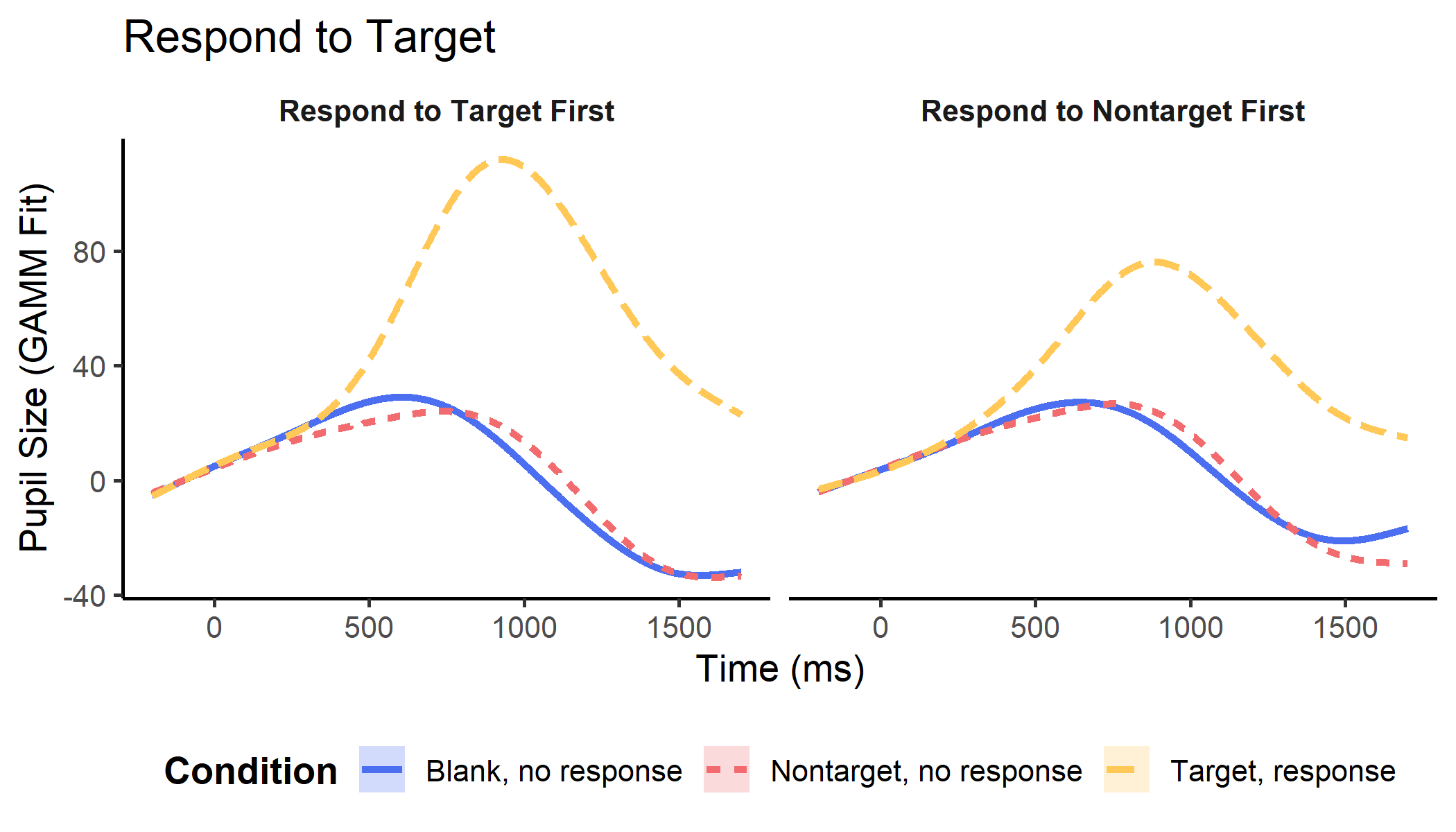
**

***Supplementary Figure 4A.*** *Generalized additive mixed model (GAMM)-predicted pupil time course during the Respond to Target block, plotted separately for participants who completed the Respond to Target block first (left panel) and those who completed the Respond to Nontarget block first (right panel). Lines show the GAMM–fitted pupil size across time for each trial condition (Blank: solid blue; Nontarget: dotted red; Target: long-dashed orange). Time 0ms corresponds to stimulus onset.*


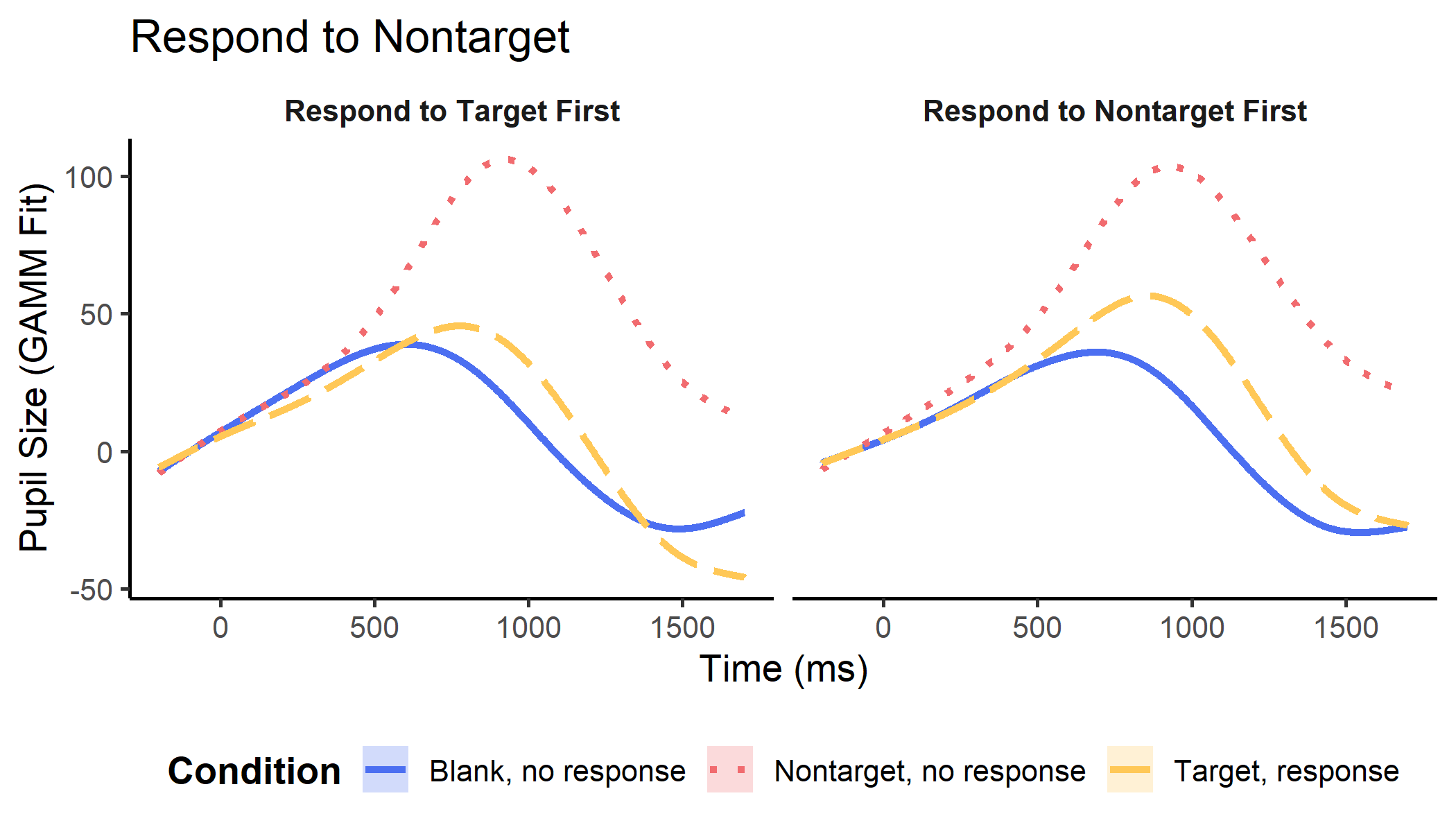


***Supplementary Figure 4B.*** *GAMM-predicted pupil time course during the Respond to Nontarget block, plotted separately for participants who completed the Respond to Target block first (left panel) and those who completed the Respond to Nontarget block first (right panel). Lines show the GAMM–fitted pupil size across time for each trial condition (Blank: solid blue; Nontarget: dotted red; Target: long-dashed orange). Time 0ms corresponds to stimulus onset.*

**Supplement 5. Memory-Pupil Index Comparison.**
To directly compare the magnitude of the boost across the Memory (Experiment 1) and Pupillary (Experiment 2) experiments, we computed parallel relative and absolute boost indices for each participant. For Respond to Target trials, the relative boost index was defined as Target – Nontarget; for Respond to Nontarget trials, it was defined as Nontarget – Target. Absolute boost indices reflected the difference between the behaviorally relevant stimulus and the blank baseline (Target – Blank or Nontarget – Blank). All indices were baseline-normalized by dividing each participant’s boost value by their own Blank-condition mean. Reported in the table are the mean (SE) of each normalized index for both experiments.

***Table Supplement 5.*** *Mean (SE) of Baseline-Normalized Boost Indices in the Memory and Pupillary Experiments*

**Respond to Target**

|  | **Memory Mean (SE)** | **Pupil Mean (SE)** |
| --- | --- | --- |
| **Absolute boost** | 0.18 (0.01) | –2.49 (3.00) |
| **Relative boost** | 0.12 (0.05) | –2.12 (3.02) |

**Respond to Nontarget**

|  | **Memory Mean (SE)** | **Pupil Mean (SE)** |
| --- | --- | --- |
| **Absolute boost** | 0.06 (0.04) | 0.62 (4.44) |
| **Relative boost** | 0.005 (0.034) | 7.73 (10.60) |
